# Modeling Climate Change Impacts on a Socioeconomically Vital Plant: The Case of *Comanthera elegans* (Goldenfoot Flower)

**DOI:** 10.1002/ece3.72031

**Published:** 2026-01-15

**Authors:** Maria Luiza de Azevedo, George Amaro, Eric Bastos Gorgens, Thiago Almeida Andrade Pinto, Fernanda de Aguiar Coelho, Débora Sampaio Mendes, Juliana Fonseca Cardoso, Ricardo Siqueira da Silva, Farzin Shabani

**Affiliations:** ^1^ Department of Forestry Engineering Federal University of Jequitinhonha and Mucuri Valleys, Campus JK Diamantina Brazil; ^2^ Embrapa Roraima Boa Vista Brazil; ^3^ Department of Agronomy Federal University of Jequitinhonha and Mucuri Valleys, Campus JK Diamantina Brazil; ^4^ Department of Ecological Modelling Helmholtz Centre for Environmental Research – UFZ Leipzig Germany; ^5^ College of Arts and Sciences, Qatar University Doha Qatar

**Keywords:** conservation, everlasting flowers, MaxEnt, species distribution models

## Abstract

*Comanthera elegans* is a threatened, endemic species of the *campos rupestres* of the Espinhaço Mountain Range—a region recognized as a biodiversity hotspot—and has great ecological and societal relevance to local traditional communities. Despite the importance of this species in these systems, the effects of climate change on its distribution remain relatively unknown. We employed the MaxEnt algorithm to model the current potential geographic distribution and the habitat suitability of this species under future climate scenarios to address this knowledge gap. We considered the SSP2‐4.5 and SSP5‐8.5 scenarios, based on four global climate models (MRI‐ESM2‐0, MIROC6, EC‐Earth3‐Veg, and CMCC‐ESM2). The model exhibited high performance, indicating a strong affinity of the species for environments with high rainfall seasonality and mild temperatures. Our models predict a substantial loss of suitable habitat for *C. elegans* under scenarios of future climate change, particularly under high greenhouse gas emission scenarios, where high‐suitability areas could be reduced by as much as 95% by 2060. Our results highlight the need for the implementation of conservation actions, including the expansion or creation of protected areas in climate refugia, alongside efforts to promote the development of cultivation techniques and regulations on harvesting practices, in order to mitigate the species' vulnerability to climate change.

## Graphical abstract

*Comanthera elegans*, an ecologically important species in rocky grasslands, is projected to face significant habitat loss under future climate change scenarios. Modeling with the Maxent algorithm showed that its suitable habitats, defined by high rainfall seasonality and moderate temperatures, are at risk, especially under high greenhouse gas emissions. These findings call for urgent conservation actions, including protected areas and sustainable management practices.

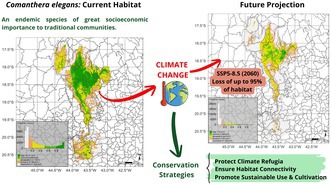

## 1 Introduction

Climate change is modifying ecosystems and putting biodiversity at risk all around the globe (Yang et al. 2022). Average global temperatures are projected to rise by 1.8°C to 4°C by 2100 (Skendžić et al. 2021). Endemic species with narrow geographic distribution and strong dependence on certain environmental conditions are among those more vulnerable to these changes (Manes et al. 2021).

Brazil is home to the world's greatest floral biodiversity and faces enormous challenges in ensuring its preservation (Forzza et al. 2012). The *campos rupestres* (rocky grasslands) are among the most threatened systems in the country, are considered biodiversity hotspots, and consist of a type of herbaceous‐shrub vegetation occurring on rocky outcrops, where about 15% of the country's plant biodiversity is found in only 1% of the territory (Silveira et al. 2016; Fernandes et al. 2020). The uniqueness of these areas is expressed in their high rate of endemism, with species adapted to a mosaic of extreme environmental conditions, such as high irradiance and predominantly sandy, shallow, and nutrient‐poor soils (Negreiros et al. 2014; Fernandes 2016). Multiple anthropogenic drivers are currently compromising the integrity of these ecological systems, such as mining activities, pasture expansion, and eucalyptus plantations (Conceição et al. 2015). This process of degradation has both ecological and social costs, as it impacts the traditional populations who rely on these ecosystems for their sustenance and the perpetuation of their cultural practices (Fernandes et al. 2020).

*Comanthera elegans* (Bong.) L.R. Parra & Giul., popularly known as “pé‐de‐ouro” (goldenfoot flower), is among the most emblematic species of the Eriocaulaceae family and one of the most commercially valuable everlasting flowers (*sempre‐vivas*) of the *campos rupestres* (Oliveira et al. 2015). This species, endemic to the Espinhaço Range, receives an endangered classification (Martinelli and Moraes 2013), having a limited geographical distribution in Minas Gerais state, Brazil. The species holds great social significance, especially for traditional communities (Parra et al. 2010). For these groups, harvesting their inflorescences represents a considerable source of income through handicraft production, while also serving as a foundation for their cultural identity, traditional ecological knowledge, and social organization (Parra et al. 2010; Fávero et al. 2021). Currently, the cultivation of *C. elegans* is still incipient, and most of its trade relies on extractivism, making the local economy dependent on the conservation of its natural habitats (Barreto et al. 2013; Fávero et al. 2021). Recognizing this unique socio‐ecological synergy, the system was designated as Brazil's first Globally Important Agricultural Heritage System (GIAHS) by the FAO–UN (Borges and Branford 2021). Previous studies have been conducted regarding its taxonomy, seed physiological quality, and the effects that harvesting can have on its populations (Nunes et al. 2008; Parra et al. 2010; Oliveira et al. 2015). Meanwhile, the potential influences of climate change on *C. elegans* distribution are poorly understood.

Species Distribution Models (SDMs) are powerful tools for identifying or predicting appropriate habitats in different climate scenarios, representing an important tool in the construction of environmental policies and species management strategies (Addison et al. 2013; Martin et al. 2020). Despite their potential, the use of SDMs to assess the vulnerability of endemic species remains limited (Qazi et al. 2022; Benavides et al. 2024) due to their restricted distributions and intricate dependence on microhabitats (Schwartz et al. 2006; Lomba et al. 2010). On the other hand, the MaxEnt algorithm stands out as one of the most effective modeling methods for this kind of scenario, maintaining the robustness of results even with small sample sizes (Hernandez et al. 2006; Wisz et al. 2008).

Based on the growing threat of climate change to biodiversity, we evaluated the effects of different climate change scenarios on the distribution of *C. elegans* , aiming to provide valuable information for its conservation. The goal was to use climatic and occurrence data to model *C. elegans* ' current potential geographic distribution via the MaxEnt algorithm and project its potential future distribution under climate change scenarios (SSPs 2–4.5 and 5–8.5). We also aimed to identify the environmental variables that most influence the species' distribution and to assess changes in the extent of suitable habitat under different climate scenarios.

## 2 Materials and Methods

### 2.1 Occurrence Data

Various sources were accessed to obtain occurrence data for *C. elegans* , including scientific articles (Oriani et al. 2009; Leal et al. 2014; Costa et al. 2016), field studies, and the Global Biodiversity Information Facility (GBIF; https://www.gbif.org/) platform, retrieved using the {rgbif} package, version 3.8.0 (Chamberlain et al. 2024). To guarantee data quality, we performed cleaning protocols based on literature recommendations, and the {bdc} and {CoordinateCleaner} packages (Zizka et al. 2019; Ribeiro et al. 2022). The process consisted of the following steps: (a) keeping only records with spatial resolution of ≤ 1 km; (b) removing records located within a 10 km radius of capital cities and 5 km of administrative centers (national, state, and municipal); (c) excluding records with identical absolute coordinates, zero values, or those located within a 0.5° radius of the GBIF headquarters; (d) removing records located in water bodies or those not associated with all selected environmental variables; (e) retaining only records within known areas of occurrence of the species.

The analysis of species distribution using presence or presence‐background data encounters sampling bias problems, which result in incorrect interpretations of occurrence–environmental predictor relationships (Barber et al. 2022; Schartel and Cao 2024). To minimize this bias, we applied a geographic filter, selecting only one occurrence per pixel, using the {flexsdm} package and the function *occfilt_geo*, based on the spatial resolution of the predictor variables used in the model (30 arc‐seconds) (Veloz 2009; Varela et al. 2014; Aiello‐Lammens et al. 2015; Velazco et al. 2022). This procedure helps prevent a sharp reduction in the available dataset.

### 2.2 Environmental Data

For modeling, we used 19 bioclimatic variables retrieved from the WorldClim version 2.1 database. The data have a spatial resolution of 30 arc‐seconds (~1 km at the equator) and provide estimates for the period from 1970 to 2000. We obtained these data using the {geodata} package, version 0.6‐2 (Hijmans 2023a), and used them to assess current (historical) climatic conditions, allowing us to capture annual variations and limiting factors known to influence species distribution (O'Donnel and Ignizio 2012). In order to simulate future climate scenarios, we utilized data from the Global Climate Models (GCMs) MRI‐ESM2‐0, MIROC6, EC‐Earth3‐Veg, and CMCC‐ESM2 under the emission scenarios SSP2‐4.5 and SSP5‐8.5 for the 2021–2040 and 2041–2060 ranges. Elevation was incorporated using SRTM (Shuttle Radar Topography Mission) data for the range from 60° N to 60° S, and GTOP30 data as a complement for latitudes above 60°. We employed four SoilGrids edaphic variables (Poggio et al. 2021), as soil characteristics can also influence plant distribution (Mkrtchian 2021). Soil variables included: acidity (pH H_2_O at 15–30 cm depth), organic carbon stock (kg m^−2^, 0–30 cm), cation exchange capacity (cmol (+) kg^−1^, 15–30 cm) and total nitrogen (g kg^−1^, 15–30 cm).

We selected the model variables through an iterative, data‐driven process in which we progressively refined the MaxEnt model. The resulting variables were then evaluated based on their biological relevance—a key criterion for determining inclusion or exclusion—ensuring that only those demonstrating both statistical significance and ecological plausibility were retained in the final model.

### 2.3 Calibration Area and Background Selection

The calibration area (CA) was defined as corresponding to the region “M” of the BAM diagram (Elith et al. 2011; Owens et al. 2013). To section this region, we applied the accessible area approach outlined in the BAM framework. This method is a theoretical benchmark in estimating space for the species. Such areas are defined by the opportunities and constraints to species movement (M); that is, places where they may potentially occur (Cooper and Soberón 2018; Mendes et al. 2020).

Model performance metrics are directly influenced by the size of the CA. Larger calibration areas generally result in improved discrimination ability (i.e., the ability to correctly distinguish presence and background locations) (Barbet‐Massin et al. 2012; Amaro et al. 2023). This occurs because larger areas often contain background points with ecological conditions that are more distinct from presence locations, which facilitates their differentiation (Lobo et al. 2008; VanDer Wal et al. 2009). However, the model's ability to predict the probability of occurrence tends to decrease as the calibration area expands, since larger extents may include regions far from presence locations that are not necessarily relevant for inferring species–environment interactions (Acevedo et al. 2012).

We defined the calibration area of the model using the biogeographic entities method (Rojas‐Soto et al. 2024). In this method, we used terrestrial ecoregions (Olson et al. 2001) as biotic units, which reflect areas with similar environmental conditions and evolutionary histories (Barve et al. 2011). Based on this, a continuous polygon was created that includes all ecoregions where the species was recorded, ensuring that the calibration area represented the species' accessible area.

We selected the background sample to reflect the relevant environmental conditions for contrasting with occurrence points, taking into account the ecological questions of interest (Saupe et al. 2012). On this matter, 10,000 points were randomly selected (Phillips and Dudík 2008; Barbet‐Massin et al. 2012), stratified to match the presence points in each partition (Hirzel and Guisan 2002). We conducted a two‐dimensional kernel density estimation sampling (Elith et al. 2010; Fitzpatrick et al. 2013; Cerasoli et al. 2017; Georgian et al. 2019, 2021; Burgos et al. 2020; Finucci et al. 2021; Robinson et al. 2021; Stephenson et al. 2021; Anderson et al. 2022), adjusted to the species occurrence coordinates, using a 0.5% bandwidth for the calibration grid area, via the *kde2d* function from the {MASS} package. This procedure also contributed to further neutralizing potential sampling bias effects.

### 2.4 Model Development

The R environment, version 4.4.0 ‘Puppy Cu’, was used to carry out all data processing, model development, map creation, and graph generation (R Core Team 2023). An automated framework, guided by best practices and recommendations from the literature, was used to model species distribution using MaxEnt (maximum entropy algorithm) (Santini et al. 2021; Srivastava et al. 2021; Rojas‐Soto et al. 2024). The spatial data analysis and transformation were performed using the {terra} package, version 1.7–78 (Hijmans 2023b) and the {sf} package, version 1.0–16 (Pebesma 2018). Variable selection for the model was performed using the {ENMeval} package, version 2.0.4 (Kass et al. 2021). The {flexsdm} package, version 1.3.4 (Velazco et al. 2022), and esources from {maxnet}, version 0.1.4 (Phillips 2021), were used for species distribution modeling procedures. ROC curve estimates and graphs for model evaluation were generated using the {pROC} package, version 1.18.5 (Robin et al. 2011). Map creation was performed using the {tmap} package, version 3.3–4 (Tennekes 2018) and publication‐quality graphs were created using {ggplot2}, version 3.5.1 (Wickham 2016).

Spatial block cross‐validation was employed to divide the training and testing data. This approach allows for the management of spatial autocorrelation and a better test of the transferability of the model compared with other types of partitioning (Valavi et al. 2019; Santini et al. 2021). Spatial partitioning of square blocks was employed using the *part_sblock* function in {flexsdm} (Velazco et al. 2019). Twenty grids were generated with resolutions ranging from 0.5 (~56 km) to 5° (~557 km), in five partitions, with at least three occurrences per partition, using all data for autocorrelation testing. The grid that met the following criteria was selected: (a) the one with the lowest spatial autocorrelation (using Moran's I); (b) the one with the highest environmental similarity (using Euclidean distance); (c) the one with the smallest difference in the number of records between the training and test sets (using standard deviation) (Velazco et al. 2019). Therefore, the data were split into five blocks, using four for training and the other for testing in each iteration. The model was run five times, with each run using a different block for testing and the remaining four for training, enabling a more robust evaluation and reducing the probability of overfitting.

The modeling in this paper was conducted using MaxEnt from the viewpoint of an inhomogeneous Poisson point process (Phillips 2008, 2017; Phillips et al. 2017). MaxEnt was chosen because it has better predictive power compared to other techniques (Helmstetter et al. 2021; Valavi et al. 2022; Ahmadi et al. 2023). MaxEnt has the added benefit of producing an output that is interpretable as a model of species' relative abundance, which can be translated into presence probabilities by applying the *cloglog* function (Peay et al. 2023).

The main MaxEnt parameters—the regularization multiplier (RM) and the feature classes (FC)—were tuned to obtain the optimal model (Elith et al. 2011; Merow et al. 2013). While the RM regulates model complexity, the feature classes define the shape of the response curves for the predictor variables. The transformations of the predictor variables (features) can be linear (L), quadratic (Q), threshold (T), hinge (H), product (P), or categorical (Merow et al. 2013). The hinge features enable a smooth model fit that resembles a generalized additive model (GAM) (Elith et al. 2010, 2011). The exclusion of product features leads to an additive model that improves interpretability but diminishes the ability to detect complex interactions (Elith et al. 2011). Initially, a MaxEnt base model was fitted using RM = 1 and FC = QH, considering the sample size (Elith et al. 2011). Based on this base model, we proceeded with a refined variable selection process. The presence and background point coordinates, along with the values of all predictor variables at those points, were derived from a 30 arc‐second resolution raster and used in the modeling process. We identified and selected the most important variables for the final model based on the results of a five‐fold random cross‐validation.

The variable selection followed a data‐driven approach, iterating through all the variables, starting with the one that contributed the most, as defined by permutation importance. The Jackknife test was employed to determine which variable to retain when we detected multicollinearity between the variables (|Spearman's coefficient| > 0.7). The variable that, when excluded, resulted in the smallest reduction in model performance was dropped according to the TSS metric (Allouche et al. 2006). This process continued until only non‐correlated variables remained (Vignali et al. 2020). The model was refined after this stage by eliminating variables with less than 2% importance, thereby reducing the risk of overfitting and improving the model's generalization power (Vignali et al. 2020). To model under new conditions (different locations or future climate), other studies suggest that it is better to select a few biologically relevant variables rather than many with uncertain effects on species distribution (Araújo and Guisan 2006; Austin and Van Niel 2011).

The final model configuration was achieved by fine‐tuning 147 models, comprising seven feature classes (FC = “L”, “H”, “LQ”, “QH”, “LQH”, “LQP”, “QHP”) and 21 values for the regularization multiplier (RM = 0.5 to 5, with increments of 0.25; 7.5 and 10), considering only the previously selected variables. We selected the best combination of hyperparameters based on the highest TSS estimate, with training‐restricted values (*clamp*) and *clog‐log* output format, representing the estimated occurrence probability between 0 and 1 (Phillips et al. 2017). To calculate the permutation importance of model variables, we used the *varImportance* function available in the {fitMaxnet} package (Wilson 2024).

Finally, to ensure model robustness, several evaluation metrics were calculated (Sofaer et al. 2019; Konowalik and Nosol 2021), taking into account threshold dependence and prevalence sensitivity (Liu et al. 2009, 2013; Leroy et al. 2018). The final optimized model was projected for Brazil, the state of Minas Gerais, and the Serra do Espinhaço Meridional region, resulting in occurrence probability maps (ranging from 0 to 1) at a resolution of 30 arc‐seconds. The model output used five predetermined probability classes that were set at the following levels: (a) unsuitable (0%–10%), (b) marginal (10%–20%), (c) moderate (20%–50%), (d) optimal (50%–80%), and (e) high (80%–100%). The area of each class was estimated to establish a reference baseline for evaluating the impacts of climate change. For future forecasts, a downscaled Global Climate Model (GCM) from the Coupled Model Intercomparison Project 6 was used, obtained from WorldClim 2.1. Considering their good performance in Brazil and Latin America (Dias and Reboita 2021; Monteverde et al. 2022; Bazzanela et al. 2024), the following four GCMs were selected: (a) MRI‐ESM2‐0 (Yukimoto et al. 2019); (b) MIROC6 (Shiogama et al. 2019); (c) EC‐Earth3‐Veg (EC‐Earth Consortium (EC‐Earth) 2019); and (d) CMCC‐ESM2 (Peano et al. 2020). Two representative greenhouse gas concentration scenarios were adopted (SSPs) from CMIP6: SSP2‐4.5 (moderate emissions) and SSP5‐8.5 (high emissions). The SSP5‐8.5 scenario, in particular, depicts a potential future in which global greenhouse gas emissions remain high, also referred to as a “business‐as‐usual” future (Riahi et al. 2011). Thus, these two future scenarios constitute the two extremes of the spectrum of potential futures (stability and high emissions) over which we can evaluate a great diversity of possible future climates. The scenarios were projected over two time horizons: 2021–2040 and 2041–2060, considered appropriate for predicting both short‐ and medium‐term conservation action planning and implementation timeframes. While the GCMs share similar functions, their projections can differ substantially throughout the 21st century (Scafetta 2023), emphasizing the need for a multi‐model consideration for a robust analysis.

## 3 Results

### 3.1 Model Performance and Variable Contribution

A total of 45 occurrences were identified through literature reviews and fieldwork; an additional 74 occurrences were obtained from the GBIF database. After the data cleaning procedure, 74 presence points were validated. Then, a geographic filter was applied, reducing this number to 61 points and ensuring consistency and accuracy, with suitable information for subsequent analyses. The model's calibration area was estimated at approximately 2,092,971 km^2^, based on the ecoregions where *C. elegans* was recorded, considering a 30 arc‐second resolution. This extent is broad enough to encompass a wide variety of environments.

The variables in this study were grouped using hierarchical cluster analysis (Figure S1), based on the correlation between them (measured by Spearman's coefficient, *ρ*), which was calculated from their values at the occurrence coordinates.

The data‐driven variable selection, performed using MaxEnt in its *default* configuration, selected the following eight variables: Bio15 (precipitation seasonality), Bio05 (maximum temperature of the warmest month), Bio03 (isothermality), Bio13 (precipitation of the wettest month), Bio18 (precipitation of the warmest quarter), TotalN (total nitrogen), CEC (cation exchange capacity), and OCS (organic carbon stock). Descriptive statistics for these variables, based on their values at the coordinates of *C. elegans* occurrences, are highlighted in Table S1.

The tuning process determined the optimal parameters of the MaxEnt model as FC = LQ and RM = 0.5. The model showed excellent performance (Table S2), with high values for True Skill Statistic (TSS = 0.85373), Boyce Index (0.95287) and Area Under the ROC Curve (AUC = 0.95747), indicating a strong ability to discriminate suitable and unsuitable areas for *C. elegans* . Low omission rates (MTP = 0.01940; 10TP = 0.08456) further support the model's capacity to accurately predict the distribution of the species while avoiding overfitting.

As visual tools that complement the analysis, the AUC and partial AUC (McClish 1989; Jiang et al. 1996) provide a clear representation of the model's performance (Figure S2). The AUC^1^ provides an overall performance metric, while the partial AUC (pAUC) at 10% allows for focused evaluation within a specific region of the ROC curve to support decision‐making. Considering the pAUC, we can assert that, among the top 10% of locations predicted with the highest probabilities of occurrence, the model correctly identified 94.6% of unsuitable areas (specificity). The model also demonstrated high sensitivity by correctly identifying 96.7% of the locations where the species is genuinely present as suitable. These findings demonstrate that the model is highly reliable in identifying critical areas, with a slight bias towards sensitivity. This indicates that the model performs consistently, minimizing omission of areas where the species genuinely occurs, which aligns well with the metrics already observed.

### 3.2 Predicted Distribution Under Current Climate Conditions

**FIGURE 1:**
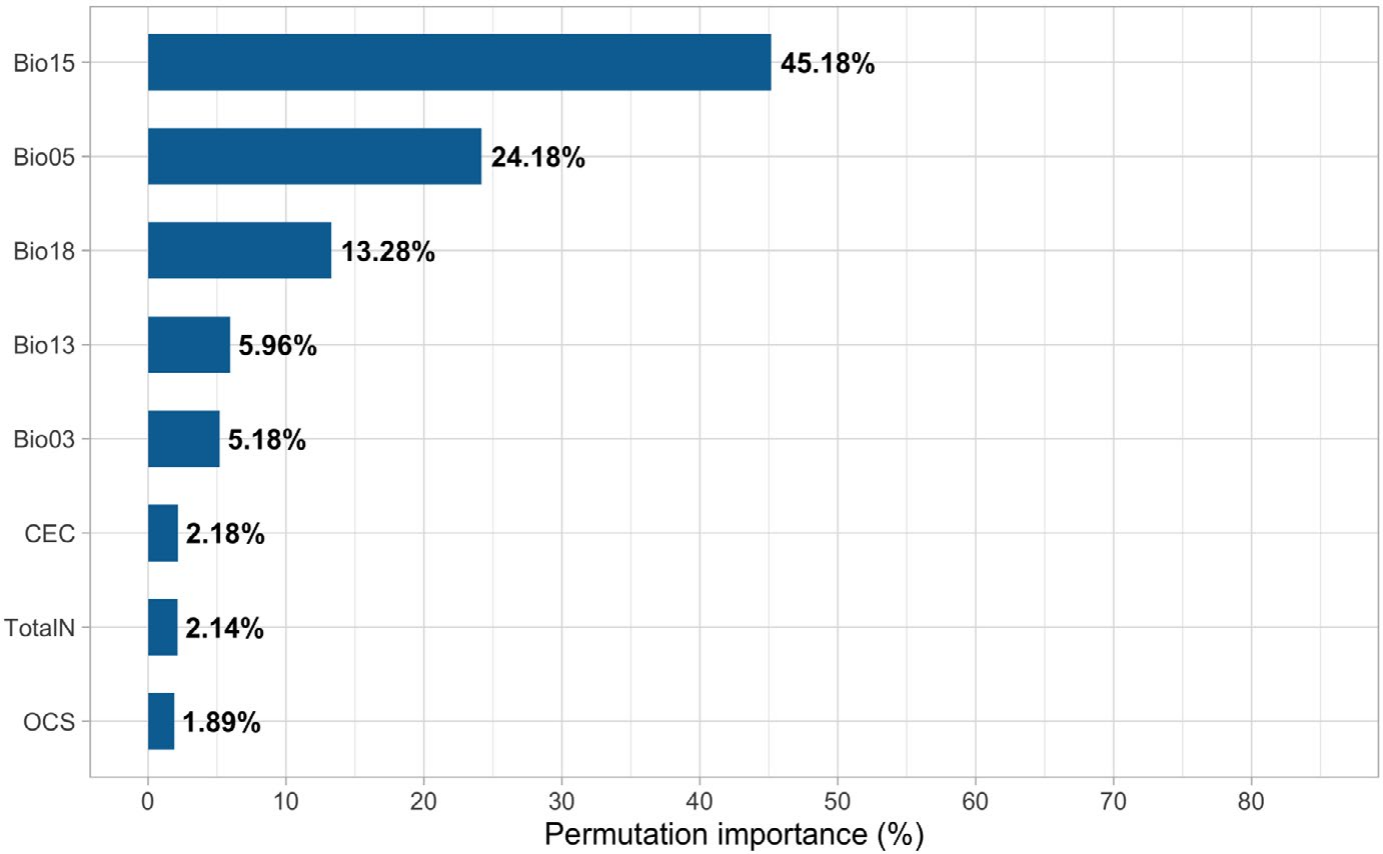
Percentage permutation importance of variables in the final MaxEnt model for *Comanthera elegans*.

In the final model, the most important predictors of *C. elegans* distribution, based on the percentage permutation of variables (Figure 1) were Bio15 (precipitation seasonality), Bio05 (maximum temperature of the warmest month), Bio18 (precipitation of the warmest quarter), and Bio13 (precipitation of the wettest month). This hierarchy of importance shows how each of the climatic factors affects the distribution of the species and gives an indication of its ecological needs, thus contributing to conservation plans.

The individual marginal response curves (partial dependence plots) illustrate the relationship between the occurrence probability of *C. elegans* and the four covariates that were of the highest permutation importance in the final model (Figure S3). These curves illustrate how the occurrence probability of the species varies with each variable when all other variables are fixed to their mean (Friedman 2001). From the response curves, we can infer that the *C. elegans* occurrence probability is high in the precipitation seasonality around 90%, indicating an adaptation to a particular hydrologic cycle. Moreover, the species prefers sites with hot month maximal temperature mild, not higher than 25°C. Precipitation in the warmest quarter, approximately 600 mm, can suggest that the species prefers a drier climate in warm months, or it might be negatively affected by rainfall abundance in such months. The species appears to be adapted to areas with moderate precipitation during the rainy season, avoiding hydrological extremes.

**FIGURE 2:**
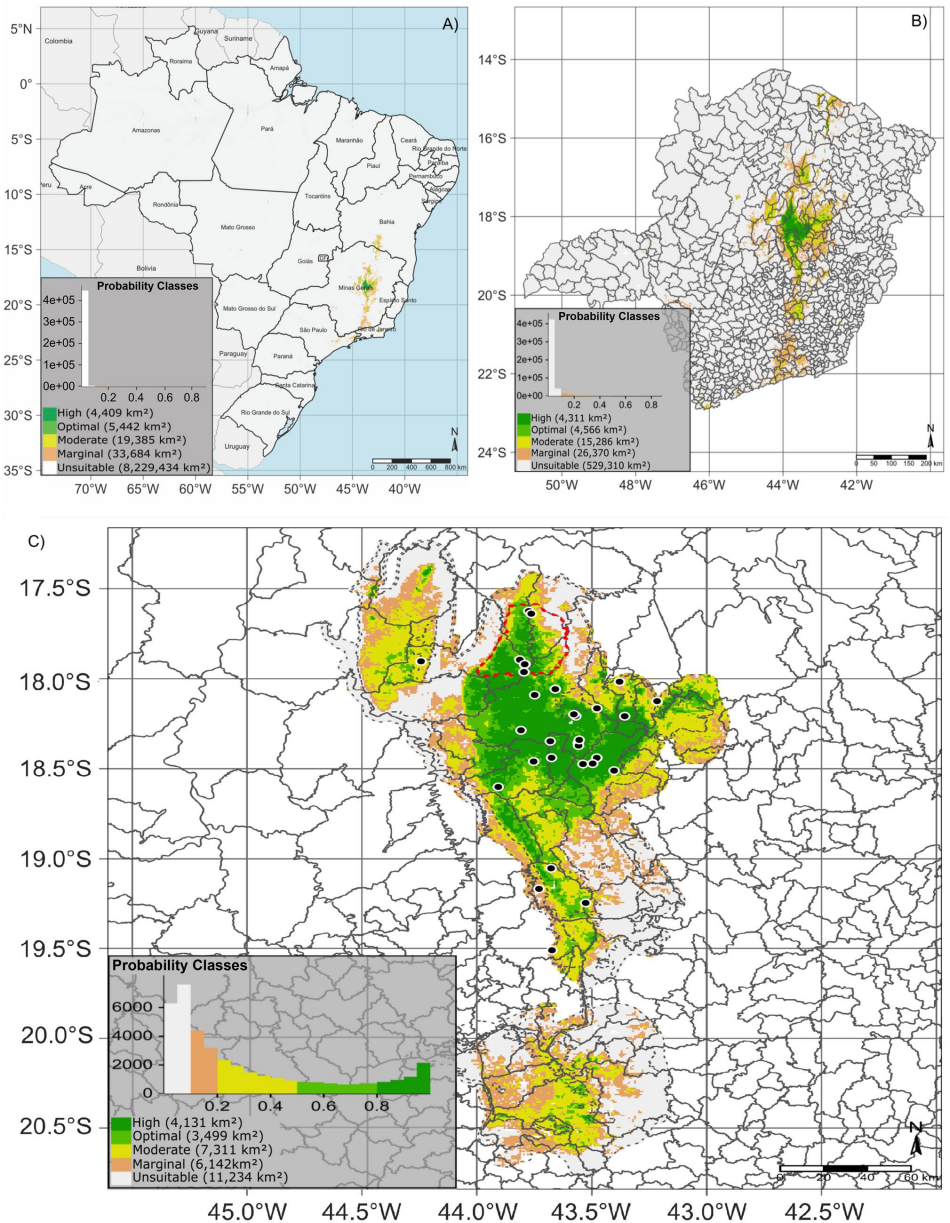
Probability classes for the potential geographic distribution of *Comanthera elegans* under current climate conditions in Brazil (A), Minas Gerais State (B), and the Serra do Espinhaço Biosphere Reserve (C). The black dots represent the species' occurrence points. Dashed in red is the Sempre‐Vivas National Park.

Figure S4 shows the potential geographic distribution of *C. elegans* under current climatic conditions. The highest probabilities are observed in the state of Minas Gerais, notably within the Serra do Espinhaço Biosphere Reserve (RBSE). The specified probability classes—unsuitable (0%–10%), marginal (10%–20%), moderate (20%–50%), optimal (50%–80%), and high (80%–100%)—along with an estimate of the corresponding areas at a 30 arc‐second resolution, can be observed in Figure 2. Environments classified with an optimal to high probability of *C. elegans* occurrence cover approximately 9000 km^2^.

### 3.3 Potential Distribution Under Future Climate Conditions

A comparison of the current and future geographic distribution of *C. elegans* , projected using the MaxEnt model and averaged across global climate models for various SSP scenarios, indicates substantial habitat loss. This reduction is most pronounced in the high‐probability occurrence classes, which represent the most suitable environments for the species' survival. This pattern underscores the species' increasing vulnerability to climate change and the urgent need for conservation strategies to ensure the protection of its critical habitat areas (Figure S5).

**FIGURE 3:**
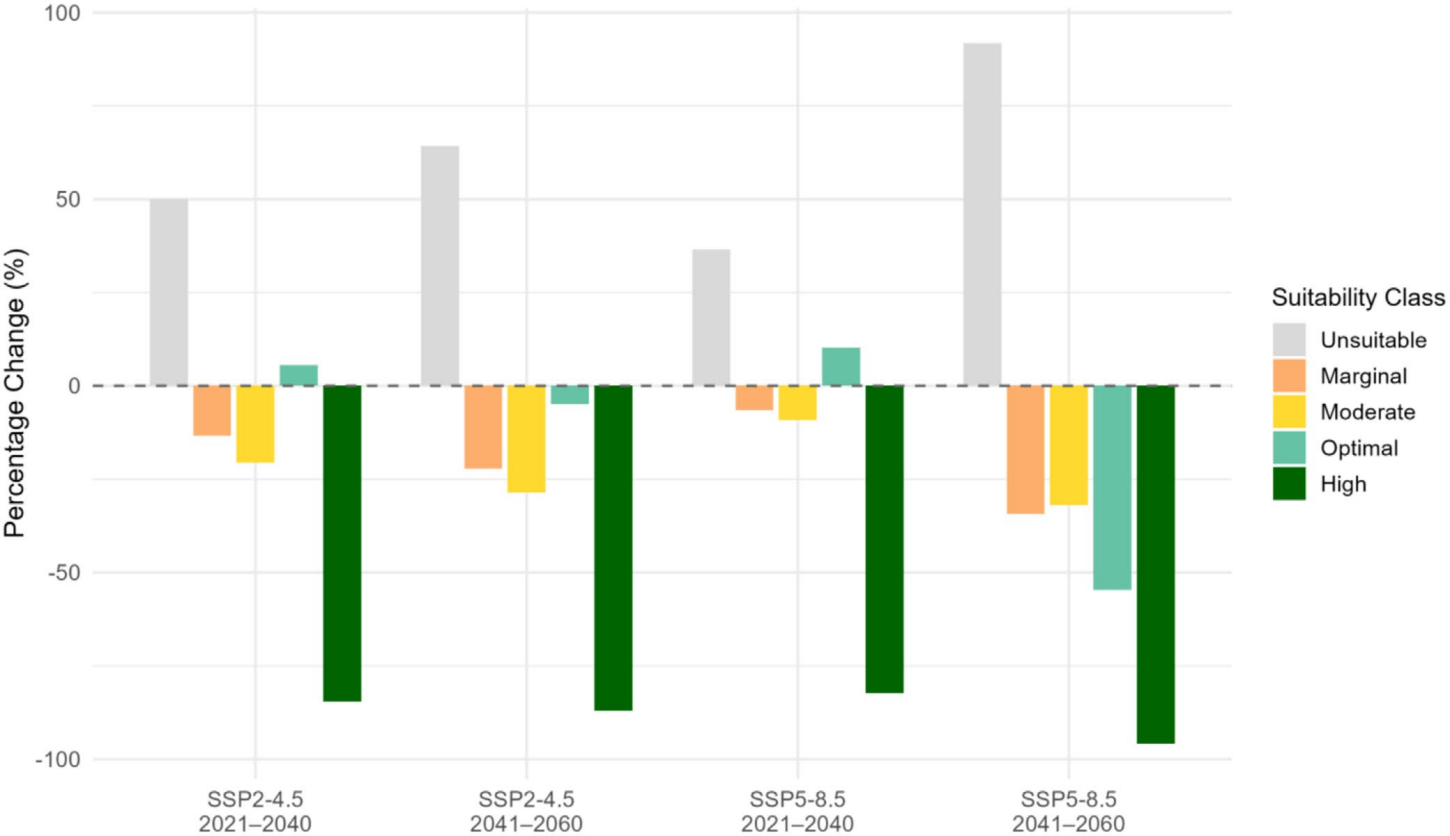
Mean percentage change in the defined probability classes across all established climate scenarios based on the estimated areas under current climate conditions, using the MaxEnt model for the potential geographic distribution of *Comanthera elegans* in the Serra do Espinhaço Biosphere Reserve (MG).

There is a pronounced reduction in high‐probability areas and an increase in unsuitable areas for the occurrence of *C. elegans* for all the GCMs, SSPs, and periods used, as well as the means of the four GCMs for each SSP and period (Figure 3; Table S3). These patterns are based on changes in the estimated areas for the occurrence probability classes, derived from a resolution of 30 arc‐seconds and classified according to the defined thresholds. This trend suggests a concerning scenario for the survival of *C. elegans* , emphasizing the need for effective monitoring and conservation measures.

## 4 Discussion

Our results indicate that the species' distributional patterns are strongly affected by precipitation‐associated variables. Specifically, the variable Bio15 (precipitation seasonality), which reflects annual variation in precipitation, showed a high influence on habitat suitability. Bio15 is calculated as the coefficient of variation of monthly precipitation, with high values indicating a significant difference between dry and rainy seasons (O'Donnel and Ignizio 2012). *C. elegans* was most likely to occur at Bio15 values close to 90%, suggesting that the species is favored in habitats with high precipitation seasonality and well‐defined dry seasons—a precipitation pattern frequently observed in *campos rupestres* (Alves et al. 2014; Fernandes 2016). Another variable that emerged as an important factor for the distribution of *C. elegans* is Bio18 (precipitation in the warmest quarter), which indicates the total amount of rain falling during the warm months of the year. The species had the greatest chance of occurring in sites with moderate precipitation in the hottest quarter (ca. 600 mm), which can be attributed to the need for water for growth and reproduction in this period without causing soil waterlogging (Moreira et al. 2017).

The *campos rupestres*, the natural habitat of *C. elegans* , show a strong seasonal climate characterized by an alternation between rainy summers and dry winters, with the dry period extending up to 5 months (Fernandes 2016). During the dry season, the *campos rupestres*' sandy, well‐drained soils are under extremely low moisture conditions, creating a challenging environment for vegetation (Nunes et al. 2016; Schaefer et al. 2016). *C. elegans* roots, as well as other species that are common in this ecosystem, are adapted to survive dry periods, including the production of mucilage secreted by epidermal cells (Scatena et al. 2005). This mucilage prevents the roots from drying out (desiccation) and increases the roots' ability to take in water and nutrients (Scatena et al. 2005). In the underground part of the everlasting flowers, there is an underground stem that stores starch, an important adaptive strategy for xerophytic conditions, which is a peculiar characteristic for the preservation of the plant's regrowth capacity (Fávero et al. 2021; Nunes et al. 2016; Schaefer et al. 2016; Fernandes 2016).

*Comanthera elegans* exists in campos rupestres at elevations higher than 900 m (Alves et al. 2014), and the humid fog represents an alternative water source, especially during dry seasons (Boanares et al. 2019). The foliar water absorption, which is facilitated by fog, is considered ecologically relevant in seasonally dry environments, where additional atmospheric water input might be a crucial factor in the maintenance of vegetation (Oliveira et al. 2014). The climate pattern, with strong seasonal variations and high‐altitude fog formation, demonstrates why Bio15 (precipitation seasonality) is important and shows how the species has adapted to obtain moisture in challenging conditions. In addition to the previously mentioned soil‐level drought strategies, the species' ability to thrive in these conditions is explained by its foliar mechanisms for absorbing atmospheric moisture. Studies show that these mechanisms include specialized anatomical features, such as trichomes (leaf hairs) that increase the surface area for water contact and mucilage within the leaf cells, which acts as a water reservoir, providing a critical water source when soil moisture is low (Boanares et al. 2018). Bio05 (maximum temperature of the hottest month) is also important, and our results indicate that the species prefers areas where the maximum temperature does not exceed 25°C. The positive correlation with precipitation in the hottest quarter (Bio18) and the moderate response to precipitation in the wettest month (Bio13) indicate the necessity of adequate moisture during specific periods.

The species depends on climatic conditions, and rising temperatures and precipitation pattern changes threaten to disrupt its sensitive ecological balance. Thus, the projections show that climate change will pose a critical threat to *C. elegans* , as its suitable habitat is expected to dramatically decrease in the future. The SSP5‐8.5 scenario predicts that high climatic suitability areas for the species will decrease by about 95% during the period from 2020 to 2060. In this context, the model developed in this study revealed that Diamantina, Serro, Couto de Magalhães de Minas, São Gonçalo do Rio Preto, Datas, Gouveia, Buenópolis, and Olhos‐d'Água in the Serra do Espinhaço region will maintain suitable climatic conditions in the future climate change scenarios. Thus, these sites should be prioritized for the implementation of conservation measures for the species.

Despite these alarming projections, it is important to acknowledge the remarkable ability of the species to survive under extreme water stress conditions, where soil moisture reaches as low as 1% during the dry season (Moreira et al. 2017). The species' capacity to survive under harsh conditions may be linked to its adaptive mechanisms, which include root mucilage production, rhizome presence (Scatena et al. 2005; Fávero et al. 2021) and other physiological adjustments (Negreiros et al. 2014). However, the projected climate changes may pose even greater challenges for *C. elegans* , which will need extra adaptations to cope with temperature increases and modified precipitation patterns. Moreover, the genetic diversity within populations may enable natural selection to favor individuals with greater tolerance to these new environmental conditions (Ledón‐Rettig et al. 2014), influencing the long‐term persistence of the species.

In addition to climate change, *C. elegans* faces many anthropogenic threats. Deforestation and habitat conversion due to agricultural and mining expansion can cause the fragmentation and destruction of its natural areas (Fernandes et al. 2014). Moreover, the unsustainable collection of inflorescences, if not adequately managed, can affect the regeneration of populations (Oliveira et al. 2015). The combined effects of these factors with climate change increase species vulnerability, thus requiring the implementation of integrated conservation strategies. This contraction in the species' distribution is particularly concerning in the Serra do Espinhaço, a global center of endemism for Eriocaulaceae, where the Sempre‐Vivas National Park (Parque Nacional das Sempre‐Vivas, PNSV) is located, housing a considerable portion of the world's everlasting flower species (Echternacht et al. 2011). The PNSV plays a crucial role in the conservation of *C. elegans* because it houses a substantial number of the species. The predicted decrease in species distribution area inside the park will result in population fragmentation, genetic isolation, and increased competition with other species. The limited dispersal capacity may hinder its migration to more suitable areas in the future, exacerbating the impacts of habitat loss (Trovó and Stützel 2011).

The predicted decline in *C. elegans* habitat is expected to have significant adverse consequences for biodiversity conservation and the livelihoods of local communities who utilize this species. The everlasting flower harvest, a practice deeply rooted in local culture, is a significant cultural and economic activity for these communities and was officially acknowledged as a GIAHS by the FAO (Borges and Branford 2021). Species distribution models, such as the one developed here, are crucial tools for predicting the impacts of climate change on species distribution and guiding actions to conserve biodiversity (McSHEA 2014; Mendes et al. 2024).

Considering the high vulnerability of *C. elegans* to climate change, conservation strategies should be established. This requires: (1) conducting population and genetic diversity monitoring in conjunction with the local community; (2) protecting climate refuges, especially high‐occurrence potential areas; (3) an integrated fire management program informed by studies into the species response to prescribed burning; and (4) promoting habitat connectivity. Furthermore, ex situ measures, such as seed banks and assisted migration to new suitable locations, are necessary for the persistence of both *C. elegans* and traditional livelihoods. The anticipated loss of suitable habitats is an indication of the risks to *C. elegans* , as well as to other endemic plants of the *campos rupestres*, from climate change. Further research is required to evaluate the impacts of climate change on other species, aiming to improve the understanding of the biodiversity threats in this ecosystem and to provide a basis for comprehensive conservation strategies.

Despite the robustness of our modeling and the relevance of our results, it is important to acknowledge the inherent limitations of species distribution models. The model only uses abiotic variables without considering potential biotic interactions (e.g., competition and pollination) and does not consider other factors inherent to the biology of *C. elegans* that limit its dispersal, which could further restrict the species' distribution. For instance, the pollination of everlasting flowers depends on small insects from the orders Hymenoptera, Diptera, Coleoptera, and Lepidoptera (de Azevedo et al. 2024). Lack or decrease in these pollinators in some regions may have negative effects on the reproductive success and distribution of this species. It is important to recognize that species distribution data, such as those used in this study, may present limitations related to sample size and sampling bias, which can affect projection accuracy (Moudrý et al. 2024). The model maintains a fixed ecological niche for *C. elegans* without considering its capacity to adapt through phenotypic plasticity or evolutionary adaptation to climate change. Moreover, there are uncertainties related to future greenhouse gas (GHG) emissions (Giorgi 2010). Finally, the development of effective management strategies also requires research on reproductive biology together with germination requirements and fire and harvesting responses (Neves et al. 2011; Oliveira et al. 2015; Bedê et al. 2018; de Azevedo et al. 2024).

## 5 Conclusion

The study provides evidence that the distribution of *C. elegans* is closely correlated with some climatic factors, such as seasonal precipitation and moderate temperatures. The research shows that predicted future climate change trajectories are expected to severely reduce the habitat suitability for this flagship species, which may lose an estimated 95% of its habitat by 2060. The severe population reduction indicates how climate change affects this species while highlighting the necessity of establishing conservation strategies.

The expected impacts require an immediate response, including the expansion of protected areas, the creation of regulations for harvest, and the promotion of sustainable cultivation. In addition, strategies to protect the species also include seed banking, in conjunction with assisted migration to projected climate refugia. These findings not only validate the importance of integrating climatic factors into conservation planning but also raise broader concerns about maintaining other endemic species exposed to the same risk in biodiversity hotspots.

## Author Contributions

**Farzin Shabani:** supervision (equal), writing – review and editing (equal). **Maria Luiza de Azevedo:** conceptualization (equal), data curation (equal), formal analysis (equal), validation (equal), visualization (equal), writing – original draft (equal). **George Amaro:** conceptualization (equal), investigation (equal), methodology (equal), writing – original draft (equal). **Eric Bastos Gorgens:** data curation (equal), formal analysis (equal), supervision (equal), validation (equal), writing – original draft (equal), writing – review and editing (equal). **Thiago Almeida Andrade Pinto:** data curation (equal), formal analysis (equal), methodology (equal), validation (equal), writing – original draft (equal), writing – review and editing (equal). **Débora Sampaio Mendes:** investigation (equal), methodology (equal), resources (equal), software (equal), visualization (equal), writing – original draft (equal), writing – review and editing (equal). **Fernanda de Aguiar Coelho:** data curation (equal), resources (equal), validation (equal), writing – original draft (equal), writing – review and editing (equal). **Juliana Fonseca Cardoso:** data curation (equal), formal analysis (equal), resources (equal), validation (equal). **Ricardo Siqueira da Silva:** data curation (equal), formal analysis (equal), investigation (equal), methodology (equal), software (equal), supervision (equal), visualization (equal), writing – original draft (equal), writing – review and editing (equal).

## Disclosure

Generative AI Statement: The authors affirm that no generative AI was employed in generating the methods, code, modeling results, figures, or tables in this work. Instead, AI tools such as ChatGPT and Grammarly were used exclusively to improve the readability, grammar, and style of the text.

## Conflicts of Interest

The authors declare no conflicts of interest.

## Supporting information


**Data S1:** ece372031‐sup‐0001‐Supinfo1.xlsx. *Comanthera elegans* Ocorrencia 2024‐08‐12.

**Data S2:** Supporting Information.

## Data Availability

The species occurrence data are provided in the Supporting Information, and all environmental data were sourced from publicly available repositories as cited in the Methods. The R code and further details can be provided by the corresponding author upon request.

## Endnotes

^1^
The AUC value in the ROC curve graph (Figure S2) differs from that presented in Table S2, though it remains within the estimated range. This discrepancy arises due to differences in calculation methods and the fact that the former is based on the mean of the Maxent model estimates, derived from the partitioning of the dataset.
