# Supplementary material for: Modeling Climate Change Impacts on a Socioeconomically Vital Plant: The Case of Comanthera elegans (Goldenfoot Flower)
Source: Ecol Evol. 2026 Jan 15;16(1):e72031. doi: 10.1002/ece3.72031 (PMC12805885; doi:10.1002/ece3.72031)
Supplement: Supplementary file 2 — Data S2: Supporting Information. [file ECE3-16-e72031-s002.zip › Supp.docx]

**Supplementary Material**


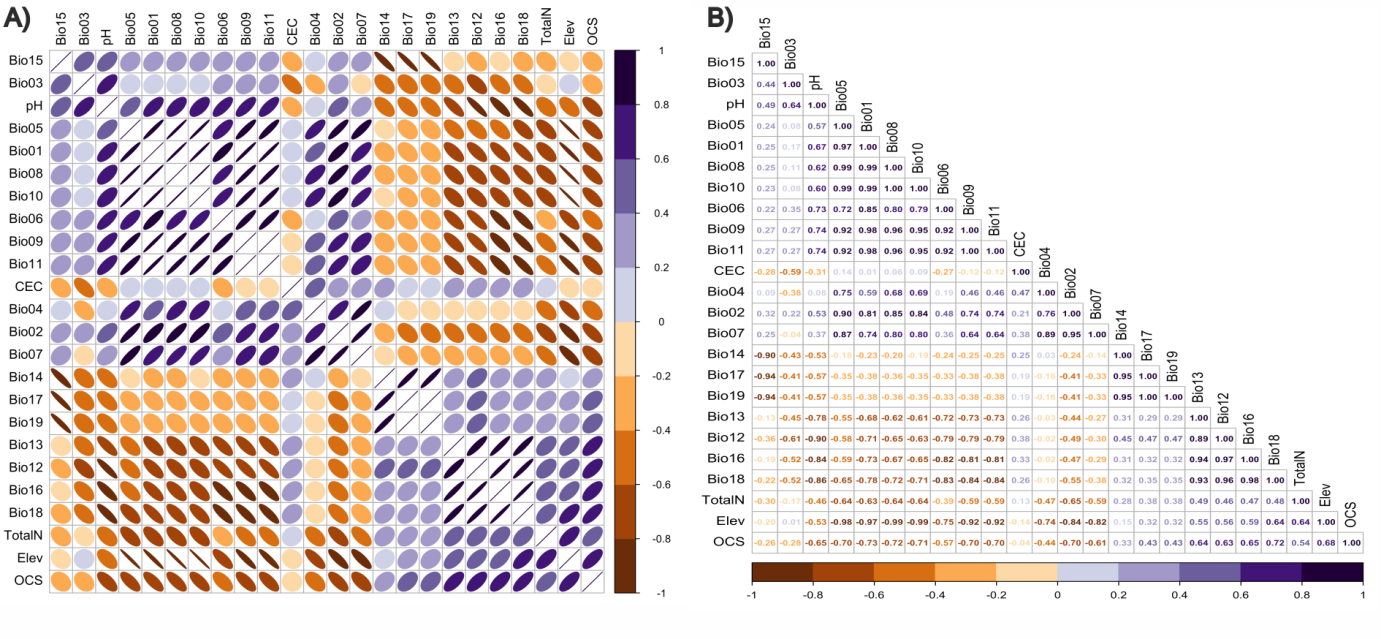
Figure S1: Spearman correlation between predictor variables and hierarchical clustering (Ward’s method). The visualization indicates: positive correlations (lilac shapes leaning to the right) and negative correlations (orange shapes leaning to the left); the intensity increases as the shape shifts from a circle (|ρ| = 0) to an ellipse (intermediate) and then to a line (|ρ| = 1). Variables were clustered using Ward’s method to form internally homogeneous and externally heterogeneous groups.


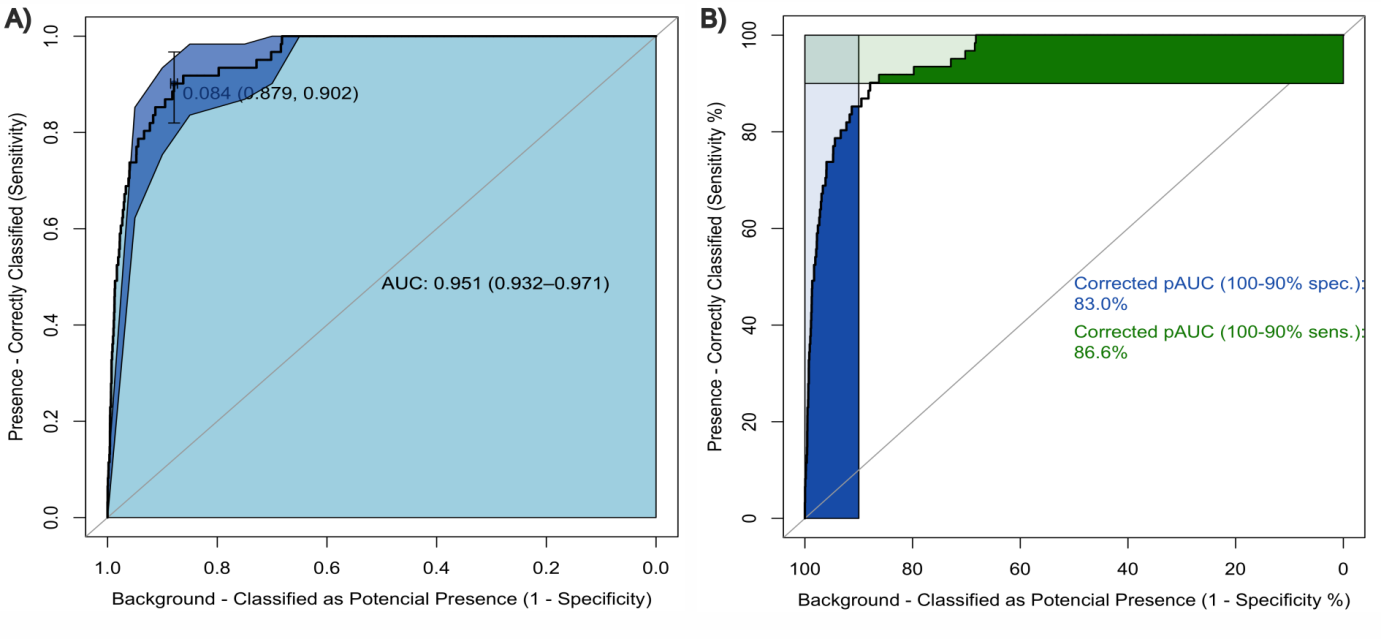
 Figure S2: Area Under the ROC Curve (AUC) (a) and partial AUC at 10% (pAUC) (b) for the final Maxent model of *Comanthera elegans*.


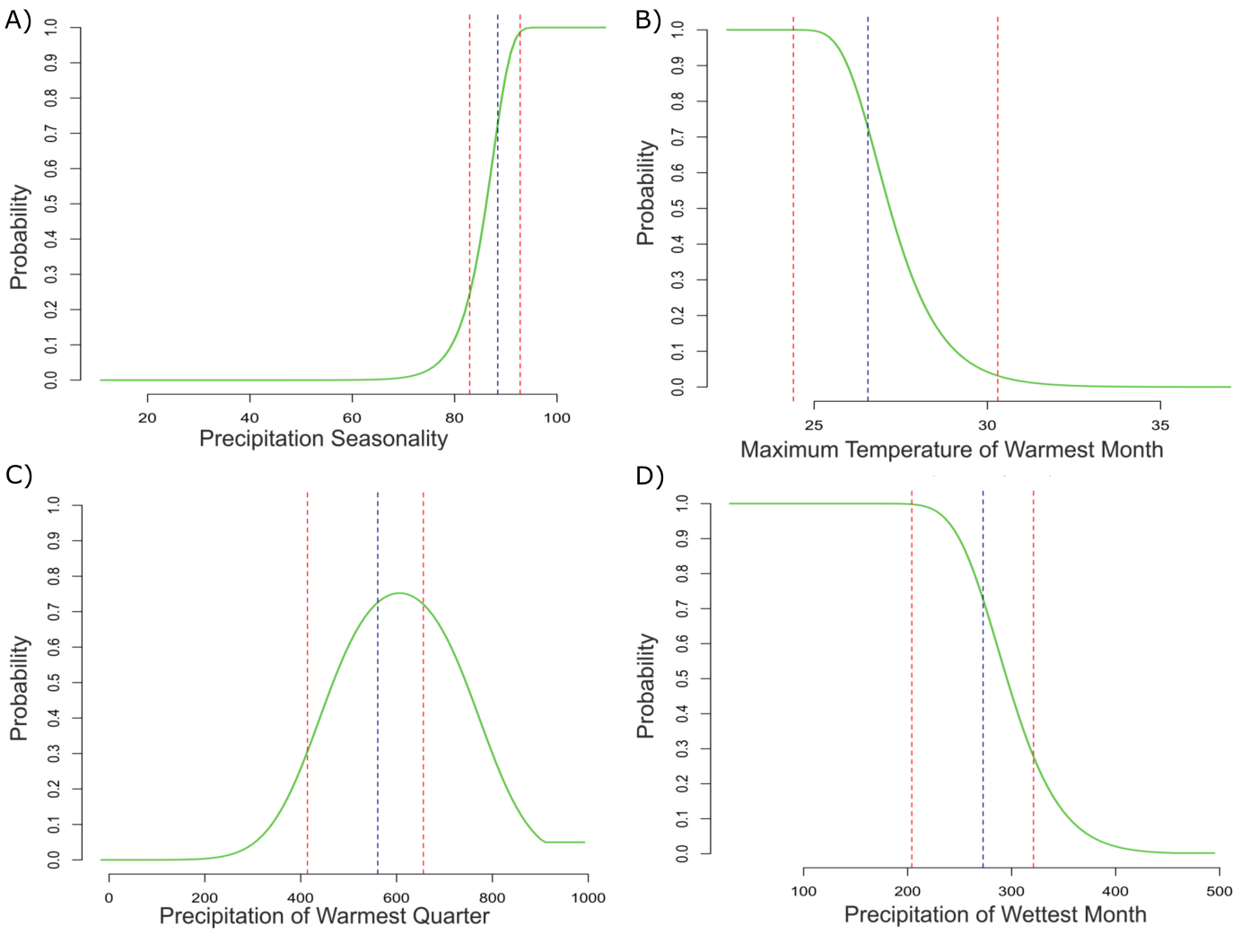


Figure S3: Individual response curves of the final MaxEnt model for *Comanthera elegans*, showing the response range (min/max, red dashed line) and the mean (dark dashed line).


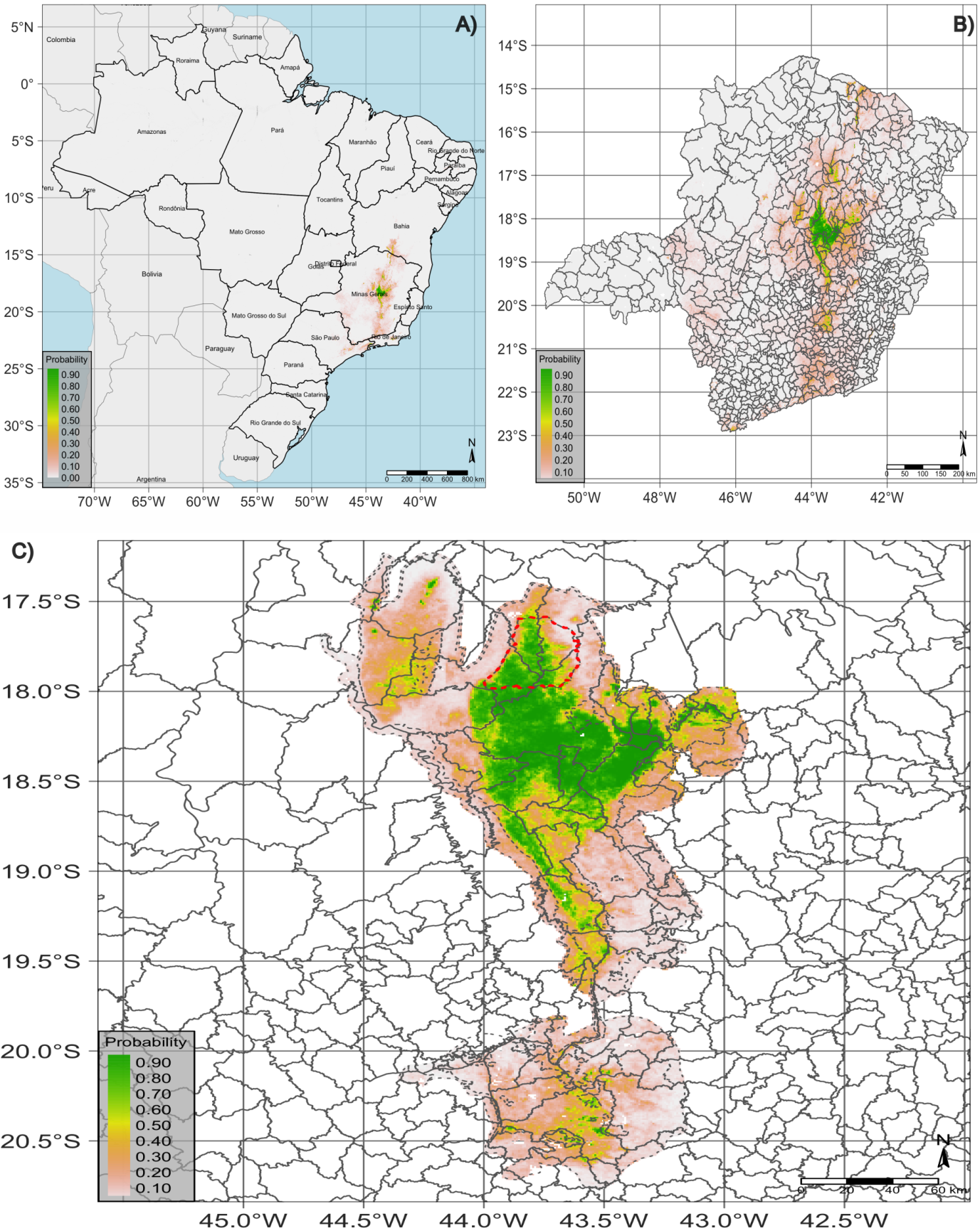


Figure S4: Potential geographic distribution of *Comanthera elegans* under Current Climate Conditions in Brazil (A), Minas Gerais State (B), and the Serra do Espinhaço Biosphere Reserve (C). Dashed in red is the Sempre-Vivas National Park.


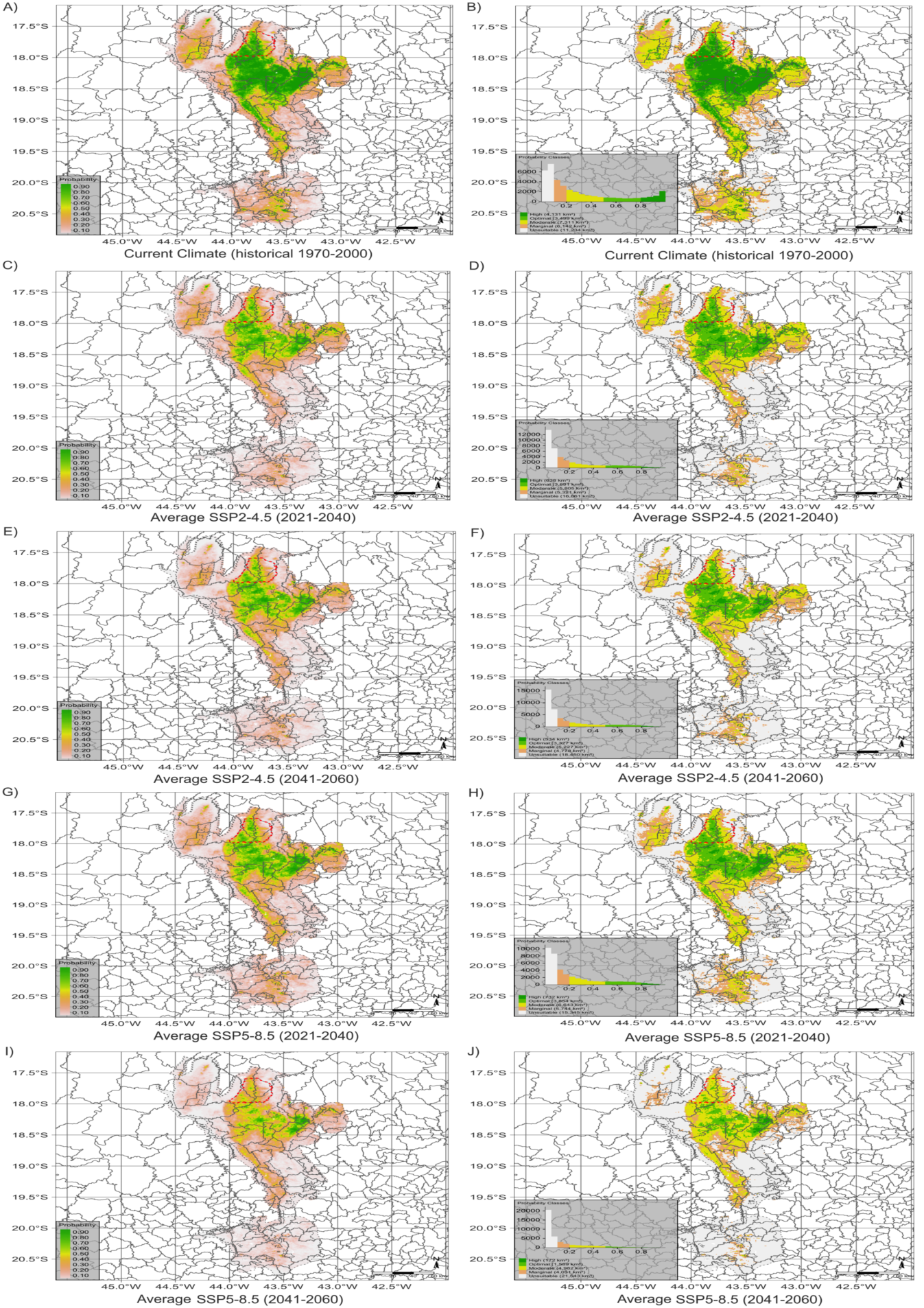


Figure S5: Potential geographic distribution of *C. elegans*, for current and future conditions, by the mean of various climate scenarios, for continuous probability (0 to 1) (a, c, e, g, i) and probability classes (b, d, f, h, j) (30” resolution), for the Serra do Espinhaço Biosphere Reserve (RBSE). Current climate conditions (1970-2000 (a, b); SSP2-4.5 scenario (2021-2040) (c, d); SSP2-4.5 scenario (2041-2060) (e, f); SSP5-8.5 scenario (2021-2040) (g, h); SSP5-8.5 scenario (2041-2060) (i, j). Dashed in red is the Sempre-Vivas National Park.

Table S1: Descriptive statistics of the models’ covariates, based on their *Comanthera elegans* occurrence coordinates values*.*

| **Variable** | **Name** | **Min.** | **Max.** | **Median** | **Mean** | **SD*** |
| --- | --- | --- | --- | --- | --- | --- |
| Bio03 | Isothermality | 63.37 | 69.02 | 65.96 | 66.12 | 1.37 |
| Bio05 | Maximum Temperature of Warmest Month | 24.40 | 30.30 | 26.00 | 26.43 | 1.50 |
| Bio13 | Precipitation of Wettest Month | 204.00 | 321.00 | 280.00 | 273.14 | 25.65 |
| Bio15 | Precipitation Seasonality | 82.93 | 92.80 | 88.25 | 88.28 | 2.13 |
| Bio18 | Precipitation of Warmest Quarter | 414.00 | 656.00 | 584.00 | 562.28 | 57.91 |
| OCS | Organic Carbon Stocks (0-30 cm) | 37.30 | 65.74 | 53.66 | 53.35 | 5.84 |
| CEC | Cation Exchange Capacity (15-30 cm) | 68.93 | 130.95 | 97.44 | 99.89 | 15.74 |
| TotalN | Total Nitrogen(15-30 cm) | 1.20 | 2.10 | 1.70 | 1.72 | 0.17 |

*SD: Standard Deviation

Table S2: Performance of the final Maxent model for *Comanthera elegans*.

| Metrics | Values |
| --- | --- |
| True Positive Rate, Sensitivity or Recall (TPR) | 0.95128 |
| True Negative Rate or Specificity (TNR) | 0.90245 |
| True Skill Statistic (TSS) | 0.85373 |
| Sorensen Index | 0.11750 |
| Jaccard Index | 0.06268 |
| F-measure on Presence-Background (FPB) | 0.12536 |
| Omission or False Negative Rate (OR) | 0.04872 |
| Boyce Index | 0.95287 |
| Area Under ROC Curve (AUC) | 0.95747 |
| Area Under Precision/Recall Curve (AUCPR) | 0.19288 |
| Inverse Mean Absolute Error (IMAE) | 0.95489 |
| False Positive Rate (FPR) | 0.09755 |
| Positive Predictive Value or Precision (PPV) | 0.90699 |
| Negative Predictive Value (NPV) | 0.48683 |
| Accuracy | 0.92687 |
| F1 Score | 0.92861 |
| Balanced Accuracy | 0.92687 |
| Matthews Correlation Coefficient (MCC) | 0.85475 |
| Minimum Training Presence (MTP) | 0.01940 |
| 10th Percentile Training Presence (10TP) | 0.08456 |
| Symmetric Extremal Dependence Index (SEDI) | 0.94453 |

Table S3: Percentage change in the defined probability classes across all established climate scenarios based on the estimated areas under current climate conditions, using the MaxEnt model for the potential geographic distribution of *Comanthera elegans* in the Serra do Espinhaço Biosphere Reserve (MG).

| **GCMs, SSPs e Periods** | **Percentage Change in Probability of Occurrence Classes** | | | | |
| --- | --- | --- | --- | --- | --- |
|  | **Unsuitable** | **Marginal** | **Moderate** | **Optimal** | **High** |
| CMIP6 CMCC-ESM2 SSP2-4.5 (2021-2040) | 62.65% | -20.16% | -11.80% | -31.78% | -92.59% |
| CMIP6 EC-Earth3-Veg SSP2-4.5 (2021-2040) | 78.69% | -37.11% | -28.03% | -23.35% | -89.47% |
| CMIP6 MIROC6 SSP2-4.5 (2021-2040) | 34.81% | -10.14% | -17.47% | -14.58% | -36.34% |
| CMIP6 MRI-ESM2-0 SSP2-4.5 (2021-2040) | 40.36% | -14.26% | -16.25% | -4.74% | -55.77% |
| **CMIP6 Mean SSP2-4.5 (2021-2040)** | **50.09%** | **-13.37%** | **-20.60%** | **5.49%** | **-84.56%** |
| CMIP6 CMCC-ESM2 SSP2-4.5 (2041-2060) | 47.91% | -17.83% | -28.52% | -8.52% | -46.11% |
| CMIP6 EC-Earth3-Veg SSP2-4.5 (2041-2060) | 101.52% | -40.72% | -33.39% | -67.91% | -98.91% |
| CMIP6 MIROC6 SSP2-4.5 (2041-2060) | 54.06% | -14.85% | -28.61% | -6.80% | -68.51% |
| CMIP6 MRI-ESM2-0 SSP2-4.5 (2041-2060) | 61.44% | -19.67% | -26.21% | -16.23% | -77.71% |
| **CMIP6 Mean SSP2-4.5 (2041-2060)** | **64.23%** | **-22.21%** | **-28.50%** | **-4.92%** | **-87.07%** |
| CMIP6 CMCC-ESM2 SSP5-8.5 (2021-2040) | 47.24% | 13.53% | 3.47% | -66.08% | -98.77% |
| CMIP6 EC-Earth3-Veg SSP5-8.5 (2021-2040) | 71.65% | -35.61% | -28.15% | -16.58% | -78.04% |
| CMIP6 MIROC6 SSP5-8.5 (2021-2040) | 29.89% | -7.33% | -15.02% | -3.77% | -40.60% |
| CMIP6 MRI-ESM2-0 SSP5-8.5 (2021-2040) | 14.14% | -8.16% | 2.41% | 0.20% | -30.77% |
| **CMIP6 Mean SSP5-8.5 (2021-2040)** | **36.59%** | **-6.48%** | **-9.14%** | **10.15%** | **-82.28%** |
| CMIP6 CMCC-ESM2 SSP5-8.5 (2041-2060) | 126.57% | -48.84% | -53.19% | -91.88% | -99.64% |
| CMIP6 EC-Earth3-Veg SSP5-8.5 (2041-2060) | 93.24% | -26.83% | -32.38% | -70.13% | -96.97% |
| CMIP6 MIROC6 SSP5-8.5 (2041-2060) | 85.08% | -33.05% | -37.83% | -34.27% | -86.27% |
| CMIP6 MRI-ESM2-0 SSP5-8.5 (2041-2060) | 66.96% | -27.89% | -26.58% | -15.60% | -80.37% |
| **CMIP6 Mean SSP5-8.5 (2041-2060)** | **91.77%** | **-34.37%** | **-31.86%** | **-54.59%** | **-95.84%** |

The negative values (-) indicates a reduction in the area.
